# Chaperone-Mediated Autophagy in the Liver: Good or Bad?

**DOI:** 10.3390/cells8111308

**Published:** 2019-10-24

**Authors:** Srikanta Dash, Yucel Aydin, Krzysztof Moroz

**Affiliations:** 1 Department of Pathology and Laboratory Medicine, Tulane University Health Sciences Center, 1430 Tulane Avenue, New Orleans, LA 70112, USA; yaydin@tulane.edu (Y.A.); kmoroz@tulane.edu (K.M.); 2 Southeast Louisiana Veterans Health Care System, 2400 Canal Street, New Orleans, LA 70119, USA

**Keywords:** hepatitis C virus (HCV), endoplasmic reticulum stress (ER stress), hepatocellular carcinoma (HCC), nuclear factor erythroid 2-related factor 2 (NRF2), chaperone-mediated autophagy (CMA), interferon alpha receptor 1 (IFNAR1)

## Abstract

Hepatitis C virus (HCV) infection triggers autophagy processes, which help clear out the dysfunctional viral and cellular components that would otherwise inhibit the virus replication. Increased cellular autophagy may kill the infected cell and terminate the infection without proper regulation. The mechanism of autophagy regulation during liver disease progression in HCV infection is unclear. The autophagy research has gained a lot of attention recently since autophagy impairment is associated with the development of hepatocellular carcinoma (HCC). Macroautophagy, microautophagy, and chaperone-mediated autophagy (CMA) are three autophagy processes involved in the lysosomal degradation and extracellular release of cytosolic cargoes under excessive stress. Autophagy processes compensate for each other during extreme endoplasmic reticulum (ER) stress to promote host and microbe survival as well as HCC development in the highly stressed microenvironment of the cirrhotic liver. This review describes the molecular details of how excessive cellular stress generated during HCV infection activates CMA to improve cell survival. The pathological implications of stress-related CMA activation resulting in the loss of hepatic innate immunity and tumor suppressors, which are most often observed among cirrhotic patients with HCC, are discussed. The oncogenic cell programming through autophagy regulation initiated by a cytoplasmic virus may facilitate our understanding of HCC mechanisms related to non-viral etiologies and metabolic conditions such as uncontrolled type II diabetes. We propose that a better understanding of how excessive cellular stress leads to cancer through autophagy modulation may allow therapeutic development and early detection of HCC.

## 1. Introduction

Hepatocellular Carcinoma (HCC) is one of the most common types of liver cancer [1]. Most of the HCC cases are accompanied by cirrhosis that results from long-standing chronic inflammation due to viral hepatitis or non-viral etiologies including heavy alcohol intake, nonalcoholic fatty liver disease (NAFLD), autoimmune hepatitis, primary biliary cirrhosis, and hemochromatosis [2]. Globally, there are significant geographic differences in HCC etiologies and trends. For more than 70% of HCC cases, the risk factors are hepatitis C virus (HCV) in developed countries and hepatitis B virus (HBV) in developing countries [3]. The remaining HCC cases (30%) are related to non-viral etiologies, including metabolic syndrome with excessive fat accumulation in the liver. Cirrhosis and HCC incidence accelerate among those who have chronic viral infection plus obesity and excessive alcohol consumption. The revolutionized antiviral treatment for HCV with the combinations of drugs called direct-acting antivirals (DAAs) is inducing a high rate of viral clearance. Successful treatment of HCV infection will prevent cirrhosis and HCC development [4]. The momentum created by the HCV cure has now renewed hope that a similar type of combinatorial treatment could be developed to cure chronic HBV infection [5,6,7]. Multiple studies have shown that HCV treatment decreased the incidence of HCC by about 70%. However, a subset of cirrhotic patients showing persistent cirrhosis after viral cure remains at risk for HCC development. The reason why these patients are still at risk for HCC following viral cure is unknown. Recently published data suggest that epigenetic alterations and loss of tumor suppressors induced by HCV infection are not reversed after DAAs therapy. Preexisting fibrosis related to alcoholic liver disease and NAFLD is a risk factor for persistent liver cirrhosis and HCC risk after HCV cure. The incidence of liver disease in the United States (US) and Western countries is expected to be shifting from viral etiologies to non-viral etiology such as NAFLD due to obesity and diabetes. In the US, NAFLD is the most common form of chronic liver disease, affecting about one-quarter of the population [8]. Metabolic conditions such as uncontrolled diabetes and dyslipidemia are some risk factors for NAFLD [9,10]. The high-calorie Western diet along with excess saturated fats, refined carbohydrates, sugar-sweetened beverages, and high fructose is also a risk factor for the development of NAFLD [11,12]. The hepatic stress response associated with excessive fat deposition in the liver leads to an inflammatory liver disease called nonalcoholic steatohepatitis (NASH). It is expected that NASH will be the leading cause of HCC in the US and Western countries in the future. The prevalence of HCC is found to be increasing progressively among the aging population, affecting males more often than females [1]. The reason for the increased HCC risk among the aging population probably relates to the multifaceted stress response associated with age-related deterioration of hepatic quality control. Therefore, a better understanding of the etiology-specific HCC mechanism will allow for early-stage detection tackling this emerging problem for effective treatment. The purpose of this review is to summarize how the hepatic stress response is connected to HCC development in cirrhosis by autophagy modulation. Since HCV infection is one of the most significant risk factors for HCC, this review summarizes the multifaceted cellular stress response generated during HCV replication in hepatocytes. 

## 2. Hepatocytes Experience Multifaceted Stress Responses during Chronic HCV Infection

Hepatocytes are the most abundant epithelial cells in the liver and have a multifaceted function that includes the metabolism of glucose, lipids, amino acids, bile secretion, bilirubin, and drug detoxification, synthesis and secretion of a large number of plasma proteins [13,14]. Hepatocytes carry an abundant endoplasmic reticulum (ER) membrane network that synthesizes many essential proteins and several lipids, including cholesterol, ceramide, phospholipids, and other functional lipids needed for the remodeling of plasma membrane, lysosomes, mitochondria, secretory vesicles, the Golgi apparatus, endosome, and peroxisomes [15]. The ER also performs protein folding and post-translational modifications such as glycosylation, disulfide bridge formation, and oligomerization. HCV is a positive-stranded RNA virus that persists entirely in the cytoplasm. The ER membranous network support continuous genome replication for HCV survival in the hepatocytes during chronic infection. The ongoing viral protein translation and accumulation as a result of genome replication causes massive rearrangement of ER membranes [16]. The excessive accumulation of viral protein with impaired protein degradation, or improper folding generates robust stress in the ER called ER stress. To survive under excessive stress, cells generate a cytoprotective gene transcription cascade called unfolded protein response (UPR) [17,18,19,20]. 

**Figure 1:**
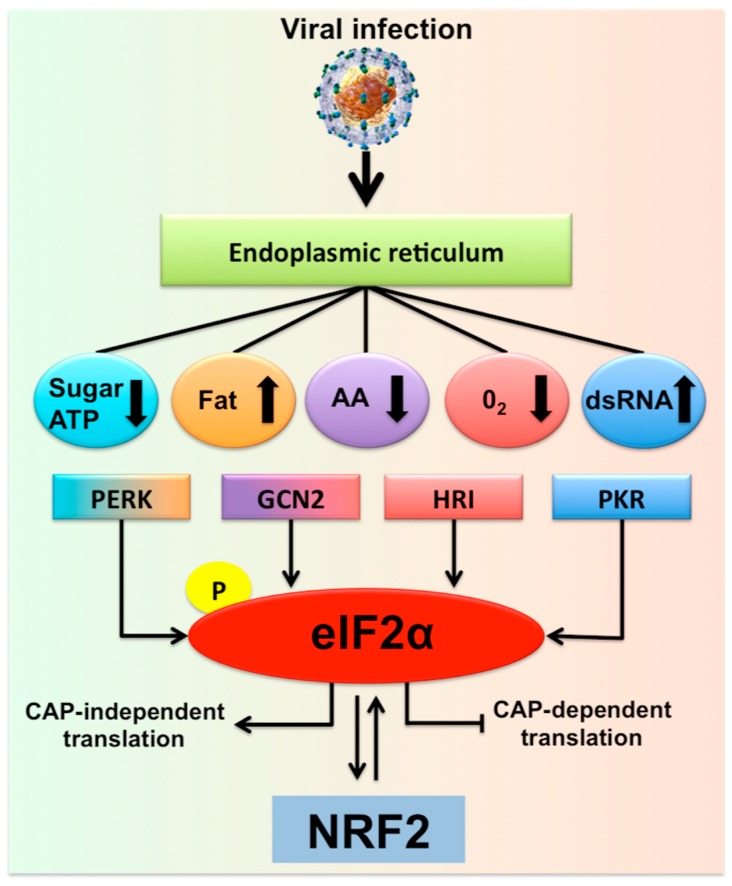
Hepatic adaptive response to multifaceted stress generated during chronic hepatitis C virus (HCV) infection is called the integrated stress response (ISR). Shown is the summary of multifaceted stress response generated during chronic HCV infection that is associated with the risk of liver disease progression. In addition to the direct virus-induced ER stress/UPR gene expression, many different cellular stress signals are induced in HCV-infected cells due to a shift in host cell metabolism. There are four different stress kinases participate in the generation of multifaceted stress response. Stress signals during HCV infection activate PKR, GCN2, PERK, and HRI kinases that stimulate phosphorylation of eIF2α, the core element of the stress response, which inhibits cellular translation. Under normal conditions with low levels of phosphorylated eIF2α promotes cap-dependent translation. During the ISR, cellular translation is attenuated due to increased eIF2α phosphorylation, which supports the translation of specific gene (ATF4) needed for cell survival. The PERK-eIF2α-ATF4 activation has been associated with autophagy induction and cell survival. If the stress becomes severe this can also activate NRF2 transcription and chaperone-mediated autophagy (CMA) activation.

The increase in cellular energy demand, as well as metabolic alterations due to the persistent virus replication, impose different types of caloric restrictions due to low levels of ATP, amino acids, sugar, and fatty acids, leading to additional stress in the hepatocytes. Infected cells adapt to various stress conditions through integrated stress response (ISR) via four different eukaryotic translation initiation factor 2 subunit alpha (eIF2α) kinases [21]. Infected hepatocytes could manage the multifaceted stress response through the induction of the ISR that promotes the transcription of numerous genes for cell survival (Figure 1). For example, viral mRNA translation requires a substantial amount of amino acid supply due to the high demand for viral protein synthesis. Hepatic amino acid depletion leads to activation of an ISR through general control non-depressible-2 protein (GCN2) and mammalian target of rapamycin complex 1 (MTORC1) pathway [22]. GCN2/eukaryotic translation initiation kinase 4(EIF2AK4) is a protein kinase that is activated in the due to cytoplasmic accumulation of tRNAs that results from amino acid depletion [23,24]. This is consistent with a report by Selitsky et al. that shows small tRNAs concentration are indeed increased in chronic HCV infection and liver cancer [25]. The low glucose concentration in the virus-infected cells imposes metabolic stress leading to a cellular energetic shift from glycolysis to mitochondrial oxidative phosphorylation that generates ATP from fatty acids and amino acids [26]. To study the hepatocellular metabolic reprogramming in HCV infection, Diamond et al. performed a study that involves an integrated proteomic and lipidomic profiling with computer modeling. These investigators have shown that HCV replication in hepatoma cells induced a metabolic shift to maintain energy demand [26,27]. The low sugar level could result in impaired glycosylation of many cellular proteins in the ER and Golgi leading to increased cellular stress through the protein kinase R (PKR)-like endoplasmic reticulum kinase (PERK)/EIF2AK3 branch of UPR [28]. Chronic HCV infection induces lipid metabolisms in the liver, leading to the accumulation of fat in hepatocytes and hepatic steatosis. To study the complex lipid metabolic remodeling in infected cells, Hoffman et al. [29] determined the lipid composition of whole cells and subcellular fractions of HCV-infected culture using a bioinformatics approach. Their study revealed that HCV infection accumulates membrane lipids, especially cholesterol and phospholipids, with a higher abundance of phosphatidylcholines and triglycerides with longer fatty acyl chains. These results are consistent with the study of Stoeck et al. [30], showing that HCV replication promotes cholesterol homeostasis. The accumulation of longer fatty acids and cholesterol in the infected cells could inhibit the ubiquitin-dependent protein degradation pathway; therefore, increases ER stress [31]. These results are in agreement with another study showing that HCV-induced cholesterol biosynthesis increases ER stress, steatohepatitis, and increases incidence HCC in HCV core transgenic mice [32]. Hypoxia (low oxygen) during viral replication creates oxidative stress, a condition generating reactive oxygen species (ROS) that promote ER stress. The oxidative stress and mitochondrial stress are mediated by heme-regulated inhibitor (HRI)/EIF2AK1 and GCN2/EIF2AK4 kinases, leading to increased phosphorylation of eIF2α. A recent review by Alfredo Rios-Ocampo et al. summarizes the intrinsic and extrinsic hepatic oxidative stress in HCV infected cell culture models and liver tissues derived from chronically infected patients reported by many laboratories [33]. PKR/EIF2AK2 is activated during viral replication by binding of double-stranded RNA (dsRNA) to its two conserved dsRNA binding domains [34,35]. The cytoplasmic stress created during persistent HCV virus replication induces DNA damage, DNA repair, and transcriptional fidelity, which contribute to genomic instability [36,37]. All these reports are indicating that a multifaceted stress response is generated during persistent replication of the HCV genome in hepatocytes.

Hepatic autophagy process is activated during HCV infection to promote cell survival and meet the energy demand due to depletion of ATP, amino acid, sugar, and fatty acids. The stress response generated from multiple sources could result in different types of cell death (necrosis, apoptosis and autophagic). If the stress signal switches from cell death to cell survival, it could lead to the emergence of malignancies like HCC. Due to these reasons, the expression of ER stress chaperones and the UPR gene is increased during chronic HCV infection, cirrhosis, and HCC. The interplay between various types of cellular stress during chronic HCV infection that causes autophagy modulation for hepatocyte survival and liver disease progression is unknown. In the following section, we describe how the infected cells survive under excessive cellular stress by modulating cellular autophagy.

## 3. Excessive Hepatic Stress Modulates Autophagy Compensation

**Figure 2:**
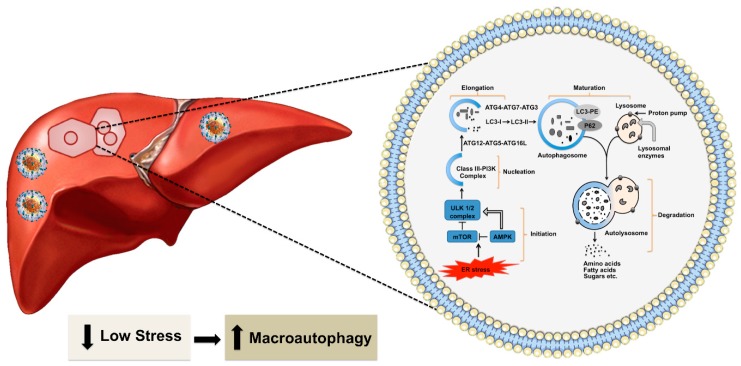
Shown is autophagy in the healthy liver. Hepatic autophagy supplies amino acids, glucose, and free fatty acids to meet the energy demand. Autophagy is activated by various stress signals derived from the ER that senses low energy state by MTOR1 and AMPK. The frequent target of these signaling pathways is the ULK complex consisting of ULK1/2, ATG13, RB1 inducible coiled-coil protein 1 (FIP200) and ATG101). This triggers the nucleation of the phagophore by phosphorylating components of the class III P13K (P13KC3) complex 1 [consisting of class III P13K, vacuolar protein sorting 34 (VPS34), beclin 1, ATG14, activating molecule in beclin 1-regulated autophagy protein 1(AMBRA1) and general vesicular transport factor p115], which activates local phosphatidylinositol-3-phosphate (PI3P) at ER membrane called omegasome. PI3P then recruits its effector proteins WD repeat domain phosphoinositide-interacting proteins (WIPIs) and Zinc-finger FYVE domain-containing protein 1 (DFCP1) to the omegasome. WIP12 binds to ATG16L directly, therefore, recruiting the ATG12-ATG5-ATG16L complex. This facilitates the ATG3-mediated conjugation of microtubule-associated protein 1 light chain 3 alpha (LC3) proteins. The conjugation reaction, which LC3-I is converted to LC3-II, is the characteristic features of autophagic membranes. Sealing of the autophagosomal membrane generates autophagosomes, which matures after fusion with endosome and then fuses with the lysosome. The acidic hydrolases in the lysosome degrade the autophagic cargo and nutrients are released back to the cytoplasm for reuse. The metabolites such as amino acids, sugars, and lipids are then released into the cytoplasm for the synthesis of new macromolecules.

During the non-pathological condition, the accumulation of misfolded proteins in the ER activates the ubiquitin-proteasome system (UPS), which is called the type I ER-assisted protein degradation (ERAD-1). This process is activated if the nutrient supply is low for a short period [38]. The type I ERAD occurs through two steps. First, damaged proteins are tagged with a ubiquitin chain, a highly conserved 76 amino acid protein, with three different enzymes (E1–E3). Second, the ubiquitin-modified proteins are then recognized and degraded by the proteasome [39]. High cellular stress response during HCV infection usually inhibits the type I ERAD [40,41,42]. If the nutrient supply is needed for an extended period during chronic viral infection, the UPR induces cellular autophagy as the second type of ER-associated protein degradation (ERAD-II) to generate energy efficiently [43,44,45]. The cascade of signals generated due to ER stress activates the autophagy pathway. Macroautophagy is widely referred to as autophagy that provides significant cellular protection under non-pathological conditions. During the process of autophagy, a part of the cytosol is sequestered in a double-membrane structure called the autophagosome, which fuses with the endosome and lysosome for degradation. This process is regulated by more than forty autophagy-related (ATG) proteins [45,46,47,48,49]. The initiation and termination of autophagy are linked to cellular nutrient sensing mechanisms and ER stress [reviewed nicely in 46]. The molecule AMP-kinase (AMPK) senses energy requirements in the cell. High AMP levels reflecting low energy status in the cell could lead to activation of the 5’-AMP-activated protein kinase (AMPK) and inactivation of MTORC1. These reactions enhance Unc-51 like autophagy activating kinase 1 (ULK1) activity. Autophagy activation follows several steps: initiation, nucleation, elongation, maturation, and degradation (Figure 2). The fusion of autophagosome-lysosome is critical for the degradation of materials by different lysosomal enzymes. The degradation products, such as amino acids, fats, and sugars, are released from the autolysosome via lysosome efflux transporters for reuse. The release of nutrients from the autolysosome reactivates mechanistic target of rapamycin kinase (MTOR), which triggers autophagy termination and the formation of nascent lysosomes. This process is called autolysosome reformation (ALR). Under conditions of low nutrition, the cycle is repeated. If the autophagy process is not controlled or remains activated for a prolonged period, it could lead to the accumulation of large-scale autophagic vacuoles in the cytoplasm that could cause cell death. Autophagic cell death occurs when a cell collapses due to depletion of the ER membrane without chromatin condensation. High-level virus replication results in autophagic cell death [50,51]. Excessive autophagy induction leading to cell death forms the basis of oncolytic virotherapy. Selected viruses (adenoviruses, herpes simplex virus, measles virus, reovirus, and Newcastle disease virus) have been used to kill tumor cells [52,53,54].

However, positive-stranded RNA viruses manipulate the autophagy pathway to develop persistent infection by overcoming the host innate and adaptive immunity [55]. To verify whether HCV induced the autophagy process in the liver, Rautou et al. [56] evaluated autophagy induction in the liver tissues of chronic HCV patients by Western blot and electron microscopy. They showed a higher number of autophagic vesicles in the chronically infected liver as compared to the standard control. To understand the clinical relevance of HCV autophagy induction in fatty liver disease, Vescovo et al. [57] examined autophagy levels in patients with HCV infection by Western blot using antibodies against microtubule-associated protein-1 light chain 3. They have found increased autophagy in HCV infected liver, but autophagy induction was inversely correlated with microvesicular steatosis due to lipid accumulation. They were able to show the co-localization of lipid droplets with autophagic vesicles. The study by Huang et al. [58] showed that HCV induced autophagy via ER stress by inhibiting AKT/MTORC1 pathway. Another study by Ke et al. [59] shows that HCV-induced ER stress/UPR activates autophagy to escape from innate immune response due to virus infection. Falco et al. [60] study demonstrated HCV associated stress response induced hepatocyte damage and ultrastructural changes, including ballooning of hepatocytes and dilatation of ER and mitochondria using liver biopsies samples from chronically infected humans. Along with a similar line of investigations, we demonstrated that ER stress and autophagy are increased during chronic HCV infection. Increased autophagy response is associated with interferon and ribavirin resistance mechanisms of chronic HCV infection [61,62,63,64]. Asselah et al. [65] demonstrated that the ER stress and UPR gene expression are increased in the liver samples from HCV-infected patients with liver fibrosis. Chandra et al. [66] study showed that the expression levels of UPR genes such as binding immunoglobulin protein (BiP), phosphorylated eIF2α, and inositol-requiring enzyme 1 (IRE1) are induced in liver biopsies of chronic HCV patients and stage IV fibrosis. The expression of activating transcription factor 6 (ATF6), x-box-binding protein 1 (XBP1), and BiP were induced in HCC shown by Shuda et al. [67], suggesting that the ER stress pathway may be involved in HCC development. The authors demonstrated that ER stress is unequivocally high in all HCC, as well as surrounding non-tumorous cirrhotic liver. The presence of ER stress/UPR activation has been reported in chronic liver diseases related to chronic HBV infection. Accumulation of mutant forms of hepatitis B surface antigen (HBsAg) in the infected hepatocytes leads to the expansion of ER and the development of ground glass hepatocytes and hepatocellular carcinoma development [68]. Chronic ER stress plays a central role in the progression of non-alcoholic fatty liver diseases due to lipid accumulation in hepatocytes [69]. The ER stress and the UPR persist during chronic liver disease and liver cirrhosis related to viral infection (HCV, HBV) and non-viral etiologies (alcohol and NASH) [70,71]. Some recent publications demonstrate the ER stress is associated with the development of liver cirrhosis and HCC [72,73,74,75]. However, the molecular mechanism for how hepatic UPR response links to HCC development is not completely understood. 

Autophagy is a lysosomal degradation process needed for energy balance and cell survival under different stress conditions, including viral infection, nutrient deprivation, hypoxia, and ischemia. Wang et al. [76] describe how the autophagy pathway is beneficial for HCV replication and persistence and cellular homeostasis. Autophagy plays a major pro-death (tumor suppressor) role in the pathogenic mechanisms of liver disease during progression from chronic liver disease to cirrhosis, and finally to hepatocellular carcinoma [77,78]. The crosstalk between autophagy and cell death (apoptosis and necrosis) in chronic liver disease balances tumor suppression and tumor progression. However, an increased number of publications have shown that the development of HCC in humans and mouse is associated with an impaired autophagy response. The first evidence of showing impaired autophagy associated with HCC was demonstrated by Yue et al. [79] using a mouse was expressing a heterozygous mutant form of beclin 1. Those mice showed an impaired autophagy process leading to a high incidence of spontaneous tumors, including liver cancer. The mutant heterozygous beclin 1 was also responsible for increasing the frequency of HBV-induced HCC [80]. This evidence suggests that beclin 1 is a tumor suppressor. A subsequent study by Takamura et al. [81] showed that the deletion of autophagy genes (*ATG5* and *ATG7*) lead to the development of benign liver adenomas in mice characterized by autophagy impairment and accumulation of p62. These authors showed simultaneous deletion of p62 reduced the size of the ATG7 knockout liver tumors. Other studies evidenced the involvement of p62 during liver tumorigenesis [82]. In particular, the accumulation of p62 was found to increase oxidative stress, reactive oxygen species, damaged mitochondria, and ER chaperones. Sustained p62 expression resulting from autophagy defects was sufficient to alter nuclear factor kappa-light-chain-enhancer of activated B cells (NF-κB) regulation and gene expression and to promote tumorigenesis [82]. A study by James Ou laboratory [83] showed that hepatocyte-specific ATG5 knockout mice developed only benign tumors with no HCC, even after treatment with diethylnitrosamine (DEN). The reason why these mice did not develop HCC with impaired autophagy because the liver of these mice shows the activation of the tumor suppressor p53. They found that the high expression of p53 suppressed the expression of NANOG, a transcription factor critical for the self-renewal and the maintenance of cancer stem cells (CSCs). The ATG knockout mouse model shows that impaired autophagy is associated with HCC development. At present, there is no evidence showing human HCC development is related to the disabled expression of ATG5 or ATG7. Another proof supporting impaired autophagy and HCC is a study of Wu et al. [84] claiming that that impaired autophagy through p62 accumulation promotes HCC through activation of cyclin D1. While all these studies support impaired autophagy link to HCC development, there are few publications showing that autophagy activation promotes HCC through the generation of cancer stem cells. One study showed that autophagy induction in the cirrhotic liver in a rat model developed HCC via activation of Axin2 expressing hepatic cancer stem cells. The cancer stem cells are induced through activation of hepatocyte growth factor (HGF)/Met/c-Jun-N-terminal kinase (JNK) signaling, and autophagy inhibition by rapamycin prevented HCC growth [85]. Another study demonstrated that the long non-coding RNAs (lncRNA) promote HCC development in the cirrhotic liver through autophagy induction [86]. They showed the overexpression of HOTAIR in HCC cell lines upregulated ATG3 and ATG7. The study of Umemura et al. [87] claims that the autophagy flux protein p62 can initiate HCC through activation of nuclear factor erythroid 2-related factor 2 (NRF2) and MTORC1 and induction of c-myc to improve cell survival of HCC under high oxidative stress. 

At present, most of the evidence connecting autophagy and HCC derived from mouse or rat models without any viral and non-viral risk factors implicated in HCC development in humans. To verify the role of autophagy in HCC development in human cirrhotic livers, we examined the expression of UPR and autophagy flux protein p62 by immunostaining. In this study, cirrhotic liver with HCC related to viral (HBV and HCV) and non-viral etiologies (alcohol and NAFLD) were included. We found that the expression levels of ER stress markers are elevated in cirrhosis and HCC related to both viral and non-viral etiologies. Increased expression of autophagy flux p62 was only found in the HCC tumor nodules suggesting that autophagy dysfunction is associated with the development of HCC related non-viral etiologies under high ER stress [88,89]. Most of the non-tumorous hepatocytes in the cirrhotic areas show negative expression of p62 protein. The mechanism of impaired autophagy, tumor cell survival and tumor growth in the highly stressed cirrhotic liver is unknown. Therefore, our laboratory is interested in understanding the mechanism through which the hepatic autophagy process promotes cell survival under high ER stress and how this sets the course of HCC development using HCV as a model system. Although ER stress is a potent inducer of autophagy, the other forms of autophagy are activated to compensate for cell survival in cases of extreme stress. In the following section, we review the molecular biology of cellular adaptive response to multifaceted stress through CMA promoted autophagy compensation and tumor cell survival. 

## 4. Chaperone-Mediated Autophagy Promotes Hepatocyte Survival under Excessive Stress

**Figure 3:**
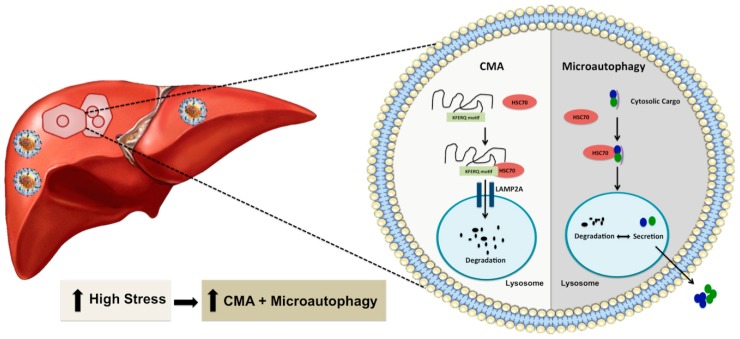
Shown is an illustration demonstrating through which excessive cellular stress at the ER activates other two forms of autophagy (microautophagy or CMA) as an efficient cellular compensatory process to reduce cellular stress and improve cell survival. CMA requires a specific cytosolic protein containing a KFERQ-like pentapeptide sequence that is recognized by HSC70 and subsequently translocated into the lysosomal lumen via interaction with LAMP2A for degradation. Microautophagy involves direct incorporation of cytoplasmic materials into endosomes or lysosomes for either degradation or export outside to reduce cellular stress. Microautophagy is also involved in the exosome secretion of cytoplasmic cargo to reduce cellular stress.

The concept of autophagy compensation during stress was originally reported by Ana Maria Cuervo group using the serum starvation model described in several publications and reviews [90,91,92,93]. One recent study by Grunvogel et al. [94] also supports such an autophagic compensatory mechanism in HCV infection. The investigators have shown that excessive HCV replicative intermediates found in the endosomal compartment and multivesicular bodies released from infected cells through extracellular vesicles. The release of cytoplasmic cargoes embedded in multivesicular bodes is required for maintaining HCV replication. Otherwise, the undegraded viral component inhibited virus replication through TLR3 signaling. Macroautophagy, microautophagy, and CMA are three well-known autophagy processes that degrade proteins, and cellular cargoes in the lysosome and endosomes. Microautophagy and CMA are two efficient cellular waste management processes that are activated when the stress response becomes severe (Figure 3). These two autophagy pathways share the same chaperone heat shock cognate 71 kDa protein (HSC70) to facilitate CMA-associated substrate degradation and endo-lysosomal release of cytosolic cargoes during microautophagy. During microautophagy, lysosomal or endosomal membranes directly internalize cytoplasmic cargoes in vesicles or internalize through HSC70 chaperone [93]. In the case of CMA, cytosolic proteins are selectively degraded in the lysosomes with the help of chaperones without any vesicle formation. CMA is highly selective because of its unique mechanism of action as compared to the two other forms of autophagy. The protein degradation by CMA occurs after translocation of the protein-chaperone complex to the lysosomes through a cascade of protein-protein interactions. Lysosomes play a significant role in CMA. Several earlier investigations have contributed to the discovery of lysosome and lysosome-mediated protein degradation. The lysosome involvement in protein degradation was discovered in the early 1950s. While investigating the mechanism of insulin action, Christian de Duve, in 1955, found that lysosomes are membrane-bound organelles that have proteolytic activities [95,96]. Subsequent ultrastructural analysis by Alex Novikoff [97] named these organelles as lysosomes. Another discovery by the Werner Strauss group revealed that lysosomes could degrade radiolabeled proteins [98]. In 1981, a liver research team headed by Nicholas LaRusso found that liver lysosomes located near the canalicular membrane of the hepatocytes are involved in bile flow and biliary lipid secretion [99]. They showed that lysosomotropic agents could reverse Ethinyl estradiol-induced cholestasis, suggesting that lysosomes are involved in bile secretion. Another group of investigators discovered that this organelle could breakdown major macromolecules, lipids, polysaccharides, and proteins due to its proteolytic and acidic environment [100,101]. Degradation of proteins by lysosomes was initially thought to be a non-specific process, but the work of Fred Dice’s group showed that lysosomes are involved in protein degradation through a selective mechanism [102]. They found that the degradation of radiolabeled ribonuclease A (RNase A) in fibroblast culture could be enhanced after serum starvation. The presence of a specific 11-amino amino acid sequence in this protein is critical for the degradation of RNase A in lysosomes [103]. This finding provided the initial clue that lysosome-based protein degradation occurs during stress related to serum starvation. The team later demonstrated that the presence of the KFERQ-like pentapeptide motif in RNase A protein promotes its degradation in the lysosome through a mechanism called CMA [103,104,105]. The cellular chaperone (HSC70) was found to bind KFERQ-like pentapeptide motifs in protein structures to carry proteins to lysosomes for degradation [106]. Another lysosomal membrane protein called lysosome-associated membrane protein type 2A (LAMP2A) was discovered as the primary lysosomal receptor for the CMA targeted proteins. Interaction of the protein-chaperone complex with the LAMP2A receptor facilitates the uptake and degradation of cytosolic protein in the lysosome [107]. The presence of HSC70 in the lysosome membrane (lys-HSC70) was found to be essential for unfolding, internalization, and degradation of proteins in the lysosome [108]. The activity of CMA is also regulated by the dynamic distribution of LAMP2A between the lysosome matrix, membrane, and changes in the monomeric form to oligomeric form [109,110,111]. Dr. Dice’s laboratory in 2000 described this lysosome-based protein degradation as CMA that is activated in various stress conditions in a mammalian system [112]. The emerging new reports claim that the CMA could be activated in other species, and the effector protein called LAMP2A was found present in birds, fish, Drosophila, and C. elegans, but absent in yeast [113,114,115,116]. 

**Figure 4:**
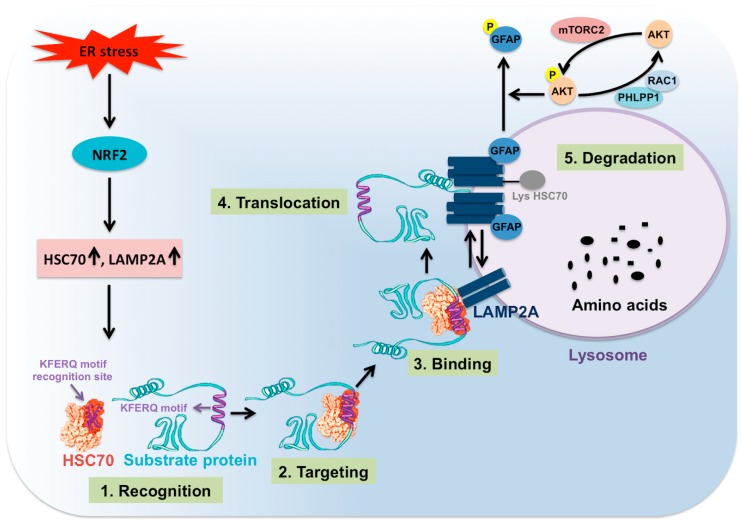
Shown are the steps involved in CMA. Cellular stress activates NRF2-related CMA, which is one of the mechanisms to improve cell survival. NRF2 induces transcription of HSC70 and LAMP2A, which are two critical molecules involved in CMA activity. CMA protein substrate containing KFERQ motifs binds to HSC70 and other chaperones. The complex binds to LAMP2A expressed on the surface of the lysosome. This promotes LAMP2A oligomerization that leads to substrate unfolding, translocation, and lysosomal degradation. GFAP favors the LAMP2A oligomerization as phosphorylation of GFAP by AKT prevents LAMP2A oligomerization and results in inhibition of CMA. Lysosomal mTORC2-mediated AKT phosphorylation inhibits CMA. PHLPP1 upregulates CMA activity through dephosphorylating AKT.

The CMA process is coordinated through multiple steps (Figure 4). The first step involves selection of specific cytosolic proteins containing pentapeptide motifs (KFERQ) for CMA degradation under stress. The specific pentapeptide motif is known as KFERQ, which has been experimentally validated to consist of a combination of one or two positively charged residues, one or two hydrophobic residues, and one negatively charged residue. The positively charged amino acid could be lysine (K) or arginine (R). The hydrophobic amino acids could be phenylalanine (F), valine (V), leucine (L) or isoleucine (I). The negatively charged amino acid could be glutamic acid (E) or aspartic acid (D). One glutamine (Q) could be on either side of the pentapeptide motif. The contributions of Ana Maria Cuervo’s group in this field have allowed us to understand many details of post-translational modifications, such as ubiquitination, phosphorylation or acetylation, that could generate the KFERQ-like motifs required for degradation by CMA [117,118]. Their analysis predicted that approximately 40% of cellular proteins were found to have this canonical KFERQ-like motif [118]. The second step is the binding and targeting of cytosolic proteins through the KFERQ motif to the lysosome surface by HSC70 cytosolic chaperone. Interaction with a number of chaperones such as heat shock protein 40 (HSP40, also known as DNANJ1), and HSP70-HSP90 organizing protein (HOP) is needed for targeting to the lysosome in an HSC70 dependent manner [118,119]. The interaction with other cochaperones such as heat shock protein 90 (HSP90) and HSP40 is required for the unfolding of the protein substrate to be degraded. The HSC70 and other cochaperones are present in the lysosome membrane (lys-HSC70), and they are absolutely necessary for CMA activity. The third step is the binding of the protein substrate to the lysosome membrane for degradation. The protein-chaperone complex binds the receptor called LAMP2A. LAMP2A is one of the three splice variants of LAMP2 (LAMP2A, LAMP2B, and LAMP2C) involved in CMA-induced protein degradation. LAMP2A has four positively charged residues in its C-terminal domain that interacts explicitly with target proteins. The fourth step involves the multimerization of LAMP2A (700kDa protein complex), leading to substrate translocation across the lysosomal membrane [117]. It has been shown that the multimerization of LAMP2A on the lysosome membrane is essential for substrate translocation and lysosome degradation [120,121]. The rate of LAMP2A assembly and disassembly after translocation of the protein complex impact the activity of CMA. LAMP2A arranges in a stable homotrimer, consisting of helical transmembrane domains bound by a coiled-coil conformation and with the cytosolic tail of this trimer bound to the complex HSC70-substrate protein [120]. The work of Cuervo’s team shows that the stability of multimeric LAMP2A on the lysosome surface is controlled by two proteins called glial fibrillary acid protein (GFAP) and eukaryotic translation elongation factor 1 alpha 1 (EF1A) [121]. The stabilizing effect of GFAP on LAMP2A oligomerization is disrupted by the association of EF1A to GTP, probably due to self-association between GFAP molecules. GFAP phosphorylation and dephosphorylation regulate CMA activity at the level of the translocation complex. The high MTORC2 level in lysosomes has been shown to inhibit CMA activity through AKT serine/threonine kinase 1 (AKT) phosphorylation. The active AKT phosphorylates GFAP, which destabilizes the LAMP2A oligomerization and substrate translocation. The high MTORC2 and AKT activation inhibit CMA activity. Pleckstrin homology (PH) domain and leucine-rich repeat protein phosphatase 1 (PHLPP1) has been shown to be recruited to the lysosome membrane by Rac family small GTPase 1 (RAC1). This PHLPP1 presence on the lysosome surface activates CMA by promoting AKT dephosphorylation. Therefore, lysosomal PHLPP1 could promote CMA activity [122,123]. The binding and uptake of the CMA substrate through the translocation complex is assisted by HSC70 and HSP90 chaperones. The fifth step involves substrate degradation and destabilization of oligomeric form to monomeric LAMP2A expression on the lysosome. HSC70 bound to the LAMP2A oligomers supports the destabilization of LAMP2A from the complex. The mechanism of CMA regulation appears to be complex and has not been established during HCV replication. This is an area where much work needs to be done to determine how the lysosomal MTORC1 and MTORC2 complexes are inversely regulated, leading to autophagy switching during excessive cellular stress in chronic HCV infection [124]. 

## 5. The Mechanism of Stress-Induced CMA

Autophagy induction benefits life cycles of a number of viruses such as HCV, dengue viruses (DENV), polioviruses, coxsackievirus B3, coronaviruses, and influenza A virus (IAV) [125,126,127]. However, excessive autophagy due to sustained virus replication and microbial stress could lead to cell death. Due to these reasons, the autophagy processes need to be regulated during pathological conditions when the cellular response is provoked by microbial stress. The persistent replication of HCV in Huh-7.5 liver cells promotes autophagy modulation through CMA activation to improve cell survival [128]. Another study shows that CMA activation is required for host-virus survival during bacteria (*Salmonella*) infection [129]. The molecular mechanism of how HCV-associated multifaceted stress activates CMA to improve cell survival is not entirely understood. The following section outlines the mechanism of CMA regulation in different cellular stress models. In addition to the CMA-related autophagy compensation, HCV can also directly inhibit autophagy through modulation of host cell protein and lysosome positioning [130].

### 5.1. Multifaceted Stress Promotes Transcription of Hsc70 and Lamp2a through Nrf2 Activation

We performed a study to understand how the excessive stress modulate autophagic pro-death to pro-survival signaling when the ER stress becomes severe or prolonged in an HCV-infected culture model [128]. Persistent HCV infection leads to the accumulation of misfolded proteins within the ER and increased expression of glucose-regulated protein 78 (GRP78). The expression of UPR genes is increased over time as a result of the hepatic adaptive mechanism to HCV-induced stress response. The key modulators of the UPR activation by virus-induced ER stress sensors, which are PERK, IRE1 and ATF6, were examined in a kinetic study over a month using HCV infection models of Huh-7.5 liver cells and primary human hepatocytes (PHHs). Among the three branches of UPR, the PERK pathway was found to be increased significantly to support cell survival during persistent HCV replication. The stress-induced PERK activation is known to phosphorylate eIF2α to decrease overall translation in order to reduce cellular stress while increasing translation of specific genes such as activating transcription factor 4 (ATF4). Activation of ATF4-C/EBP homologous protein (CHOP) pathway could induce expression of genes involved in apoptotic cell death during prolonged ER stress. The PERK activation could also directly phosphorylate the transcription factor, NRF2, which induces the expression of numerous cell survival pathways [131]. Indeed, we found that persistent HCV replication activates NRF2 phosphorylation and nuclear translocation in 100% of the infected cell. These results suggested that NRF2 activation occurs in HCV-infected cells as an alternative cell survival mechanism under prolonged stress [132]. The NRF2 activation occurs by canonical (ROS-dependent, KEAP1-dependent), and non-canonical (p62-dependent) mechanisms [133,134,135,136,137,138]. We found that *NRF2* mRNA transcription and phosphorylation are controlled by the PERK axis of the UPR in the HCV infection model, suggesting the PERK pathway could directly contribute to cell survival through NRF2 phosphorylation. Cytosolic protein uptake and degradation in the lysosome through CMA requires coordination between HSC70 and LAMP2A. We examined whether NRF2 transcription factor directly regulates the expression of CMA regulators. The presence of multiple antioxidant response elements (ARE) (TGAnnnnGC) and ARE-like (TGAnnnGC or TGAnnnnnGC) binding sites were found in the promoter region of *LAMP2A* and *HSC70* genes. This observation led us to examine whether *HSC70* and *LAMP2A* mRNA transcription are regulated through NRF2 in HCV culture. Indeed, the mRNA and protein levels of HSC70 and LAMP2A were induced in Huh-7.5 cells and primary human hepatocytes after HCV infection. The expression of HSC70 and LAMP2A was decreased after NRF2 silencing. The viability of infected cells was decreased after either NRF2 or LAMP2A silencing, suggesting that NRF2-related CMA activation is required to improve cell survival in HCV culture. This is consistent with previous reports claiming that excessive cellular stress could promote autophagy compensation, although one of the autophagy processes needs to be compromised [139,140,141]. We showed that CMA activation inhibits macroautophagy through the degradation of beclin 1. The CMA-induced beclin 1 degradation shutdown autophagy at the level of initiation and autophagosome-lysosome fusion. Our study provides an explanation of how HCV induced CMA activation to modulate autophagy pathways for improving cell survival under the extreme stressful condition of chronic HCV infection through NRF2 activation. The NRF2 pathway modulates glucose and glutamine metabolisms through more efficient anabolic pathways for improving cell survival and tumor growth under stress [142].

### 5.2. Oxidative Stress Promotes Nrf2-Mediated Lamp2a Activation 

In the same year, another publication by Pajares, et al. [143] addressed the CMA mechanism under oxidative stress using NRF2-knockout mouse model and knockout cells. They found that the *LAMP2A* gene expression is regulated by the NRF2-dependent manner since there are three AREs binding sites located in the LAMP2A gene promoter. The study showed that NRF2 binds to the AREs elements in the LAMP2A gene by ChIP assay and regulates the expression of luciferase genes from the LAMP2A promoter. The effect of lentivirus-mediated expression of NRF2 on the appearance of antioxidant genes (*HMOX1, SQSTM1, NADPH*) was examined using wild type mouse hepatocytes and NRF2-KO cells. Lentivirus-mediated expression of NRF2 in NRF2-KO cells increased *LAMP2A* mRNA but not LAMP2B or LAMP2C. The study also verified these results using wild type and NRF2-KO cells of human astrocytes, mouse hippocampal, embryo fibroblasts, and cortical neurons. Induction of oxidative stress by a small molecule drug, paraquat, and by hydrogen peroxide showed increased LAMP2A expression in wild type hepatocytes but not NRF2-KO mouse hepatocytes. A pharmacological activator of NRF2, sulforaphane, induced CMA through LAMP2A expression in wild type cells but not in the NRF2-KO cells. The CMA activity was impaired in the NRF2-KO mouse model, and degradation of GAPDH, a CMA substrate, was not decreased in KO-mice after CMA induction. The NRF2-associated expression of LAMP2A was varied using the mouse and human hepatocytes, mouse embryonic fibroblasts, neuroblastoma cells, mouse hippocampus-derived cells, human kidney cells, suggesting that NRF2-mediated LAMP2A expression is universally regulated. These data are consistent with our previous publications, suggesting that CMA activity is regulated by the NRF2 antioxidative response under stress. 

### 5.3. Regulation of CMA by Direct Phosphorylation of Lamp2a in A Non-Viral ER Stress Model

Another recent publication by Li et al. [144] shows that ER stressor could activate CMA by promoting direct phosphorylation of LAMP2A on the lysosome surface. They showed that ER stress induction by calcium pump inhibitor thapsigargin and N-glycosylation suppressor tunicamycin promoted CMA activation and decreased the expression level of a CMA targeted transcription factor called myocyte enhancer 2D (MEF2D). The study showed that ER stress increased the expression of LAMP2A and its oligomerization in lysosome without altering mRNA levels. The mechanism underlying CMA activation and LAMP2A oligomerization were found to be mediated by p38 MAPK, an essential kinase activated by ER stress. This analysis showed p38 MAPK promotes phosphorylation of LAMP2A at T211 and T213 amino acids. The loss of CMA activation leads to ER stress-induced cell death. This study showed that the PERK axis, but not the IRE1 or ATF6, is responsible for CMA activation under various stress inducers. The ER stress response utilizes the p38 MAPK-CMA pathway for cell survival under stress, thus avoiding neuronal death in a neurotoxin-induced model of Parkinson’s disease. The CMA regulation by this mechanism needs to be examined using a model for chronic viral and non-viral liver disease models. 

### 5.4. The MTOR Axis Modulates CMA through Autolysosome Lysosome Reformation

**Figure 5:**
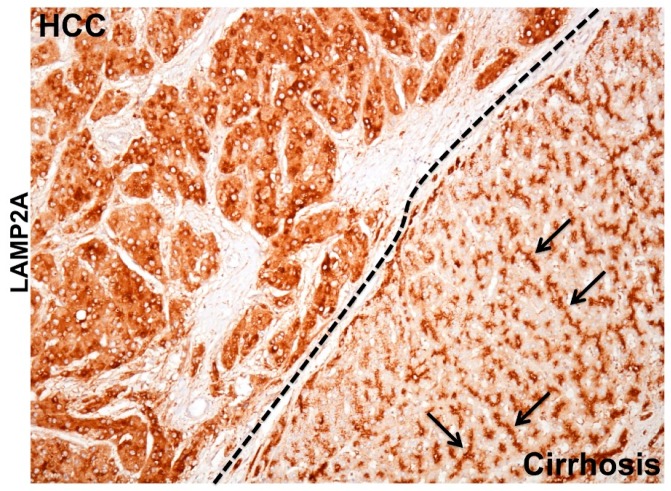
Shown is the immunohistochemical staining demonstrating remarkable differences in the pattern of LAMP2A staining between hepatocellular carcinoma (HCC) and surrounding non-tumorous hepatocytes in the cirrhotic liver. The LAMP2A staining in the HCC tumor is mostly cytoplasmic probably due to lysosome amplification or LAMP2A transcription indicating increased CMA activity. Arrows shows the bile canalicular accentuation in the non-tumorous cirrhotic liver.

MTOR is a serine/threonine kinase that functions as a critical energy sensor promoting cellular growth and cell survival under stressful conditions [145]. MTOR has two distinct complexes MTORC1 and MTORC2 that regulate two important kinases: S6K and AKT. MTORC1 and MTORC2 have been found on the lysosomal surface, and they have an opposing effect on cellular autophagy. The high-level lysosomal MTORC1 inhibits macroautophagy but promotes lysosome reformation that favors CMA [146]. On the other hand, the high lysosomal MTORC2 inhibits CMA through AKT phosphorylation that inhibits assembly of LAMP2A via GFAP phosphorylation [147]. From these reports, one could assume that low mTORC1 and high MTORC2 state is considered as a tumor suppressor since this favors high macroautophagy on the background of low CMA. On the other hand, high MTORC1 and low MTORC2 state favors tumor growth as this condition favors high CMA on the background of impaired macroautophagy. During autophagosome-lysosome reformation (ALR), lysosome-like tubules emerge from autolysosomes to form proto-lysosomes that then mature into lysosomes to enable autophagic flux [148]. It has been observed that increased cellular stress response related to prolonged serum starvation causes CMA activation where low MTORC2 promotes LAMP2A assembly on the lysosome membrane. We made a similar observation in human cirrhotic liver with HCC. High macroautophagy on the background of low CMA provides tumor protection in cirrhotic liver whereas HCC lesions show a high CMA activation on the background of impaired macroautophagy. In the cirrhotic liver, the expression of LAMP2A was exclusively localized to bile canaliculi, whereas tumor cells adjacent to the cirrhotic nodule showed strong LAMP2A staining in the cytoplasm, suggesting that the activation of CMA in HCC is also associated with increased lysosome number (Figure 5). The presence of ISR may represent a key mechanism for HCV persistence and carcinogenesis. The MTOR kinases (MTORC1 and MTORC2) are both presents on the lysosomal surface and control lysosome maintenance and proliferation. The MTOR pathway seems to play an important role in cellular autophagy through modulating lysosome activity as a platform for metabolic sensing during excessive stress [145]. This is a new area of future research that may allow critical therapeutic targets for HCC and biomarker development.

## 6. The Pathological Implication of CMA Activation in HCV-Induced Liver Disease

Autophagy is a cellular process that improves cell survival under various types of cellular stress, and autophagy impairment has been implicated in the development of human malignancy. However, the cancer development related to impaired autophagy has not been consistent in every human malignancy [149]. One possible explanation may be that the underlying cause of cellular stress, autophagy regulation, chronic disease, and cancer mechanisms are not uniform in every tissues and organs. The low cellular stress could still maintain active autophagy in the tumor whereas high cellular stress could inhibit autophagy. Autophagy plays a vital role in liver disease progression related to the HCV-induced HCC development. Autophagy levels increased several folds higher in HCV-infected individuals to deal with the stress response provoked by the virus, which may explain why the incidence of HCC is higher due to chronic HCV infection. Increased ER stress response and autophagy have been demonstrated in chronic liver disease associated with HBV [150] and HCV infection [151], alcoholic liver diseases [152], and NASH [153,154]. In most of the patients, HCC develops on the background of chronic liver diseases and is closely associated with cirrhosis. The mechanism of HCC development in the highly stressed cirrhotic microenvironment is unknown. Impaired autophagy has been described in HCC related to viral and non-viral etiologies, but the mechanisms of autophagy regulation leading to cancer are still unknown. We discussed below how the consequences of the adaptive cellular response to HCV infection through CMA activation could lead to autophagy inhibition, activation of oncogenic signaling, loss of tumor suppressor, and impairment of hepatic immunity. All these events set the stage for HCC development in cirrhosis.

### 6.1. Cellular Adaptive Response to HCV Infection Promotes Cm-Associated Autophagy Compensation Favoring HCC Growth in the Cirrhotic Liver

**Figure 6:**
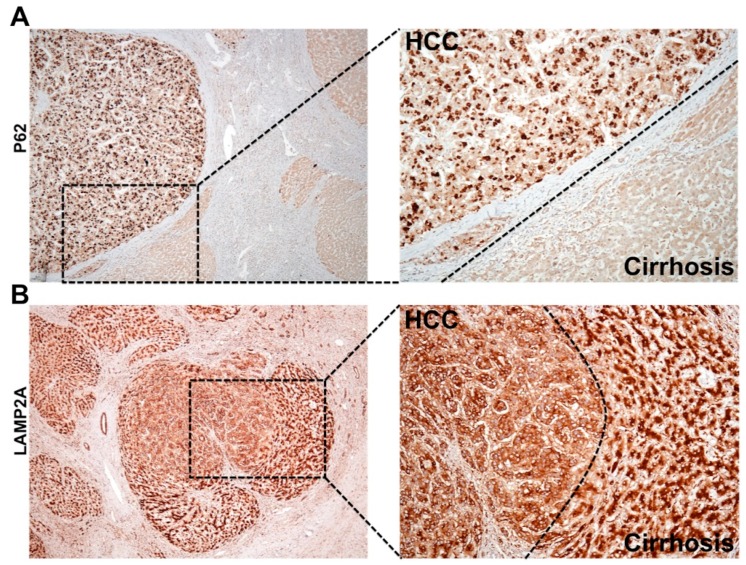
Shown is the immunohistochemical staining pattern of p62 and LAMP2A in HCC and surrounding non-tumor areas of the cirrhotic livers. (**A**). The appearance of p62 is significantly higher in HCC as compared to the adjacent non-tumorous cirrhotic liver, suggesting that HCC has impaired autophagy response. High-power micrograph shows cytoplasmic accumulation of p62 only in the HCC, but not the surrounding non-tumorous hepatocytes in the cirrhotic liver. (**B**). LAMP2A staining of HCC tumor nodule developed in cirrhotic areas. The LAMP2A staining is different between HCC tumors in the center and hepatocytes in surrounding non-tumorous cirrhotic areas. High-power micrograph clearly shows cytoplasmic LAMP2A staining in the tumor nodules, suggesting that CMA is activated in HCC.

During chronic liver disease, autophagy acts as a tumor suppressor (pro-death), and most of the HCC in the cirrhotic liver show impaired autophagy. Autophagy defects contributing to HCC development was reported initially in human liver by Ding et al., 2008 [155]. This hypothesis was verified using beclin 1, ATG knockout mouse models described earlier [79,80,81,82,83]. The autophagy status in HCC and surrounding non-tumorous cirrhotic livers was confirmed using immunohistochemical staining (Figure 6A). We found that most of the HCC tumor nodules showed increased expression of p62 and glypican 3, and the surrounding non-transformed hepatocytes were negative [88,89]. To determine whether the upregulation of CMA is involved in cancer development and growth, Kon et al. [156] examined lung, skin, stomach, colon, uterus, and ovarian cancer by immunostaining for LAMP2A. This was the first report showing CMA was especially activated in the tumor areas as compared to healthy non-tumorous tissues. Another study by Ding et al. [157] also showed CMA activation is required for HCC growth and recurrence of HCC using the tumor xenograft model in mice. The mechanism of autophagy compensation between HCC and cirrhotic areas was verified using HCV-infected liver tissue samples by immunohistochemical staining for LAMP2A and p62 [89]. A picture was showing autophagy compensation in HCC tumor nodules developed in a cirrhotic liver (Figure 6B). Increased cytoplasmic expression of LAMP2A was found only in the HCC nodule formed in the cirrhotic liver. Histochemical staining of LAMP2A showed a well-organized staining pattern with bile canalicular accentuation. The LAMP2A staining was completely cytoplasmic in the tumor nodule of the cirrhotic liver consistent with the findings of Kon et al [156]. A strong cytoplasmic LAMP2A staining is an indication of lysosome proliferation that may be used as mechanisms of increased cellular degradation to meet the energy demand to sustain HCC growth in the cirrhotic liver. These results support the hypothesis that autophagy functions as a pro-death (tumor suppressor) during chronic infection in the cirrhotic liver, but functions as pro-survival (oncogenic) during tumor formation and growth in the cirrhotic liver. The molecular and cellular mechanism of autophagy regulation under stress is unknown. Aydin et al. study [128] addressed the mechanism of autophagy compensation using a persistently HCV infected cell culture model. It was found that adaptive cellular response to HCV infection activates CMA to improve cell survival. It has been shown that beclin 1 is required for autophagy initiation and autophagosome-lysosome fusion [158,159,160,161]. The stress adaptive CMA activation in HCV-infected culture leads to beclin 1 degradation and accumulation of autophagy flux (p62) due to impaired autophagy. This study provides an explanation of why an adaptive cellular response to HCV-induced stress inhibits autophagy leading to the accumulation of p62. This is supported by clinical data showing that the impaired autophagy in human HCC is associated with decreased expression of beclin 1, and the loss of beclin 1 expression in HCC relates to poor prognosis [162,163]. These results indicate that regulated beclin 1 expression is critical for autophagy impairment in HCC. Although the accumulation of p62 due to autophagy impairment, and NRF2 activation is associated with HCC development, the mechanism of how tumor cells survive under the impairment of autophagic degradation is unclear. Taken together, all these studies are in agreement with ours, presenting a piece of strong evidence suggesting that impaired autophagy and activation of CMA is associated with HCC development in humans. In addition to the CMA activation, the NRF2 activation could initiate a Warburg effect, oncogenic driven metabolic rewiring implicated in glucose uptake and glycolysis as alternative pathways adapted for tumor cell survival in the stressed cirrhotic liver [164,165,166,167,168,169,170]. 

### 6.2. Autophagy Inhibition Activates Oncogenic Signaling

Receptor tyrosine kinases (RTKs) and cyclin-dependent kinases (CDKs) are implicated in HCC growth; therefore, they have been used as therapeutic targets for HCC treatment [171]. The mechanism of how these kinases are activated in HCC is not well established. Growth-factor-responsive RTKs have been found to play a major role in generating malignant transformation [172,173]. The activation of RTK can occur through transcriptional levels, chromosome translocation, point mutation, and improper recycling [174,175]. Among these, defective endocytosis and recycling of RTK produce the long-term mitogenic effect of growth factors by stabilizing the expression of plasma membrane receptors [176,177,178,179]. We demonstrated that the impaired autophagosome-lysosome fusion due to CMA activation in HCV culture activated epidermal growth factor receptor (EGFR) signaling by inhibiting endocytosis and degradation of EGFR. The HCV-induced impaired autophagy response could inhibit the degradation of EGFR at the level of autophagosome-lysosome fusion leading to the activation of downstream RAS/RAF/MEK/ERK signaling [128]. The activation of oncogenic signaling enables mitogenic signaling that support HCC growth in the cirrhotic liver. The autophagy inhibition related to an adaptive cellular response to virus infection could lead to the activation of the significant RTK pathway and HCC development [180,181]. EGFR is a transmembrane receptor tyrosine kinase that is overexpressed in 68% of human HCCs and correlates with aggressive tumors and patient survival [182,183,184,185,186]. The stress-induced impaired autophagy could provide growth advantage through membrane-bound tyrosine kinase receptors such as EGFR, insulin-like growth factor receptor (IGFR), endothelial growth factor receptor (IGFR), platelet-derived growth factor receptor (PDGFR) and could lead to activation of RAS/RAF/MEK/ERK signaling, increased cell growth and differentiation, and eventually to cancer. Cyclins and CDKs are ubiquitinated and degraded through the proteasomal pathway to maintain the cell-cycle progression. Wu et al. [84] study shows that autophagic degradation machinery is a cell-cycle regulator that maintains cyclin D1 expression in normal hepatocytes through proteasomal degradation through p62 mediated recruitment of ubiquitinated cyclin D1. Impaired autophagy leads to p62 accumulation and activation of cyclin D1 leading to HCC development in the mouse model. These data indicate that impaired autophagosome-lysosome fusion could activate oncogenic signaling that can promote HCC cell survival due to insufficient autophagy.

### 6.3. Hepatic Adaptive Response to HCV-Associated Stress Degrades Tumor Suppressors

**Figure 7:**
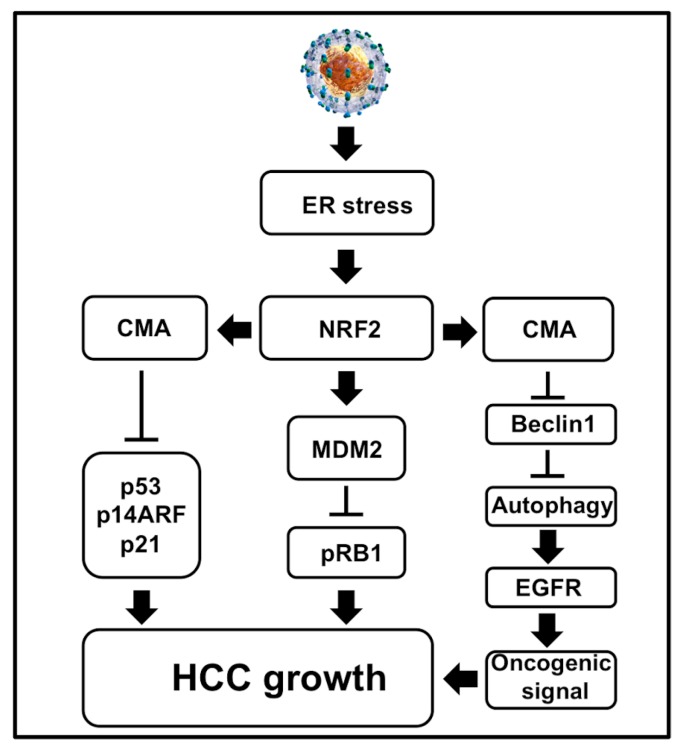
Shown is a schematic diagram illustrating how cellular adaptive response to HCV-induced ER stress results in degradation of the major tumor suppressors and autophagy inhibition. Autophagy inhibition activates oncogenic signaling at the membrane due to impaired EGFR degradation. The simultaneous loss of tumor suppressors p53, pRB, p21 and p14ARF results in cell growth and proliferation of HCC development in the cirrhotic livers. All these events promote HCC growth in liver cirrhosis.

The oncogenic mechanisms of tumor viruses involve targeting the tumor suppressors: p53 and pRB1 that control cell death and proliferation [187]. The evidence of viral hijacking tumor suppressor genes was initially observed in 1979 while studying cellular transformation by simian vacuolating virus 40 (SV40) [188]. Cellular p53 is induced during cellular stress to regulate many pathways, such as maintaining genome stability through DNA repair, cell cycle arrest, apoptosis, senescence, and autophagy [189]. Since then, many publications showed that virus replication and bacterial infection (*H. Pylori*) could degrade p53 and pRB1 for cell survival [reviewed nicely in [190,191]. There are many molecular interactions that connect these two tumor suppressors for protecting infected cells from inactivating them simultaneously [192]. Inactivation of both the tumor suppressors provides accelerated growth advantage and initiation of tumor formation. Many earlier publications studied the interactions between viral proteins (core, NS3, and NS5A) with p53. These studies were not consistent since they were not performed using infectious cell culture models [193]. The inactivation of pRB1 tumor suppressor by HCV was initially reported by Stanley Lemon’s group [194,195] using the infectious cell culture model. They showed that viral protein NS5B induces proteasomal degradation of pRB1 tumor suppressor in infected cell cultures. Since HCC developed due to viral and non-viral etiologies, we studied inactivation of p53 and pRB1 tumor suppressors in the context of cellular stress. A study by Vakifahmetoglu-Norberg et al. [196] demonstrated that the mutant p53 protein could undergo lysosomal degradation under cellular stress through CMA. Based on this publication, we examined whether the hepatic adaptive response to HCV-associated stress in the HCV cell culture model resulted in the inactivation of both the tumor suppressors, which is related to the activation of CMA under excessive stress (Figure 7). Using the HCV infection model persistently in Huh-7.5 and PHHs, we showed that the adaptive cellular response to HCV-induced stress leads to p53 degradation [197]. More specifically, we showed that CMA targeted the degradation of both mutant and wild type p53 under excessive stress. The hepatic p53 expression increased during the early stage of infection, but increased stress during the late stage of infection to promote the degradation of p53 in infected Huh-7.5 cells and primary human hepatocytes (PHHs). The p53 protein is a target of CMA because it harbors two pentapeptide motifs (200 NLRVE204 and 341FRELN345) that are similar to the HSC70 recognition sequence. Due to the presence of such pentapeptide motifs, this tumor suppressor is selected for degradation under excessive stress. Liver cells cultured under similar conditions without the virus showed stable expression of p53, suggesting that the degradation of p53 was not related to stress response associated with in vitro cell culture conditions. The appearance of p53 is controlled by cellular antagonist mouse double minute 2 homolog (MDM2) through E3-ubiquitin ligase activity. We showed in our publication that cellular stress associated with HCV infection promotes expression of the MDM2 protein through the NRF2-dependent mechanism [198]. In the HCV model, the MDM2 protein degrades pRB1 expression, not p53 expression. We found that Nutlin3 and siRNA-mediated silencing of MDM2 restored pRB1 expression, not the p53 expression. During persistent HCV infection, p53 degradation occurs mostly in lysosome by the CMA pathway. The loss of p53 expression in HCV culture could be restored by either ER stress inhibitor or a lysosome inhibitor. LAMP2A silencing prevented the degradation of p53. The alternative reading frame (ARF) protein is also another tumor suppressor that inhibits the function of MDM2, leading to p53 stabilization [198]. The adaptive cellular response to HCV-associated stress leads to degradation of p14ARF by CMA, which is consistent with an earlier report showing LAMP2A mediated p14ARF degradation in the lysosome [198,199]. Our observation is compatible with a recent publication showing that the tumor suppressor p14ARF undergoes lysosomal degradation under stress through the HSC70-dependent manner [200]. The cell culture data are also verified by measuring the expression of p53 using in HCV-infected explant cirrhotic livers. We showed that the appearance of the ER stress chaperone (GRP78), CMA-related proteins (HSC70, LAMP2A), and MDM2 were highly elevated in tissue extracts of the cirrhotic liver with HCV infection [198]. The pathway regulation by the adaptive cellular response to provoke stress due to HCV lead to loss of tumor suppressor shown in Figure 7. The observation made in a cell culture model of HCV infection is consistent with whole-genome sequencing data of many cancer driver genes altered in HCV-induced HCC [201,202]. 

### 6.4. CMA Targets Interferon-Alpha Receptor Chain-1 (Ifnar1) Degradation

It is known that HCV inhibits hepatic innate antiviral response leading to the establishment of chronic infection. This laboratory demonstrated that persistent HCV infection, as well as HCV cultured with free fatty acids, selectively blocks type I interferon (IFN) signaling [64]. The HCV-induced stress results in selective degradation of IFNAR1 by CMA. We found that there is a CMA consensus amino acid motif (QKVEV) present at amino acid 34 in the human IFNAR1 protein, and this motif is absent in the IFN-γ receptor or IFN-λ receptor. We found that IFNAR1 expression was significantly diminished after 4 hours of serum starvation and there was change on the expression of IFN-λ receptor. The expression of IFNAR1 levels was decreased in a concentration-dependent manner in Huh-7.5 cells treated with 6-aminonicotinamide (6-AN) by Western blotting. Silencing LAMP2A by siRNA rescued the IFNAR1 expression under the serum starvation condition. Since the LAMP2A serves as a receptor for the selective uptake and degradation of IFNAR1-HSC70 complex, we verified their interaction with co-IP experiments. These results now support that the activation of CMA in HCV infection leads to the degradation of IFNAR1. The expression of IFNAR1 is severely impaired in advanced liver diseases shown by Western blot analysis by our group previously [66]. This data may explain why patients with cirrhosis often develop resistance to IFN-based antiviral therapy for HCV infection used earlier. Innate immunity related to type I IFN is known to mediate antitumor response against several cancers [203]. IFNs provide an antitumor response through the elimination of tumor pre-malignant cells and malignant cells by the immune system [204]. Tissue-specific loss of IFNAR1 from intestinal cells results in increased tumor incidence in mice treated by colitis inducing agents [205]. Type I IFN also support cytotoxicity of T cells by promoting perforin and granzyme B expression and could inactivate the function of regulatory T cells [206,207]. Type I IFN also promotes cross-priming of antigens by dendritic cells [208,209]. IFN gene transcription provides the first line of defense against viral infection and is essential for restoring host innate and adaptive immunity during HCV infection [210]. These observations provide an explanation through which hepatic stress-mediated loss of IFNAR1 could result in impaired innate and adaptive immune response in the liver.

### 6.5. CMA Dampens Innate Hepatic Immunity by Preventing the P53-Ifnar1 Feedback Loop

**Figure 8:**
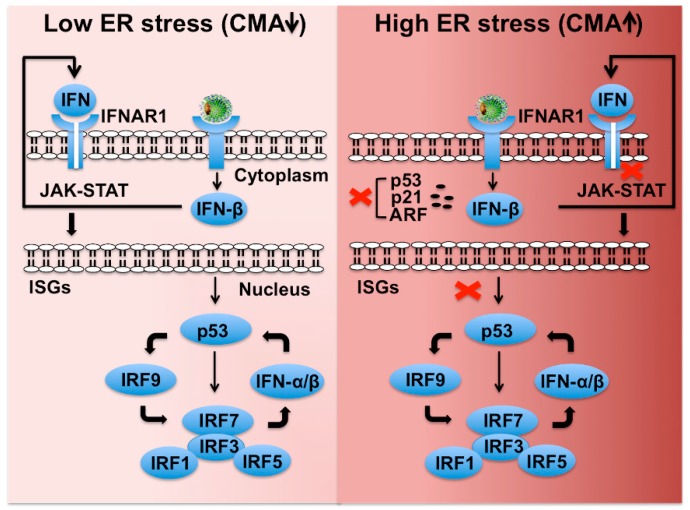
Shown is a hypothetical model demonstrating how HCV-induced severe stress response impairs IFNAR1-p53-mediated innate antiviral loop that blocks transcription of type I IFN, ISGs, and IRFs. Low stress favors cellular p53-mediated cellular expression of ISGs and various IRFs that maintain the innate hepatic immunity and cellular apoptosis. Hepatic adaptive response to virus-associated stress selectively promotes CMA-associated degradation of IFNAR1, p53, and p14ARF and favors cell survival. Loss of IFNAR1 and p53 disables the activation of the innate feedback loop leading to severe impairment of innate immunity in the highly stressed cirrhotic liver.

There are three IFN families (type I, II, III), which signal through unique receptors, but it is unclear which members of these families are actually important for hepatic response to chronic HCV infection. Type III IFN (IFN-λ 1–4) regulates similar sets of genes as type I IFN and induces antiviral state and viral clearance in the liver [211]. The transcription of type I and type III IFN is also mediated by IFN regulatory factors (IRF3, IRF7), and toll like receptor 3 (TLR3) in response to virus infection [212,213]. The p53 tumor suppressor restores innate antiviral immunity by amplifying the intrahepatic IFN signaling [214,215,216,217]. Based on our published data, we provided a model of how p53 plays an important role in the intrahepatic transcription and amplification of type I and III IFN genes through IRF transcription (Figure 8). The transcription of IFN genes operates in two steps. First, IRF3 is activated by virus infection and enters into the nucleus to produce IFN-β gene transcription. Second, endogenously produced IFN-β binds to the IFN-α/β receptor leading to the activation of the JAK-STAT pathway, leading to numerous IFN-stimulated gene (ISG) inductions. IRF7 is one of the ISGs that translocate into the nucleus to activate IFN-β and IFN-α genes. It is proposed that HCV infection also induces transcription of IFN-λ genes through a similar mechanism. Since the p53 promoter contains a functional IFN-sensitive response element (ISRE), it is IFN inducible. The tumor suppressor p53 enhances IFN signaling of host cells through direct induction of IRF9 and IRF7. Several additional ISGs, including IRF5, ISG15, and TLRs, are also p53 target genes [215,216]. We showed that HCV infection degrades p53 and IFNAR1 in favor of their continued survival under stressful conditions [64,197]. The tumor suppressor p53 is also a direct transcriptional target of type I IFNs because of the presence of ISRE elements in its promoter [215]. Several IRFs (IRF1, IRF3, IRF5, IRF7, IRF9), ISG15, and TLR3 are all induced by p53 [214,215,216,217]. Based on this information, we propose that severe hepatic stress due to persistent HCV infection escapes host innate immune surveillance through preventing the p53-, and IFNAR1-mediated amplification of innate antiviral program that results in impaired expression of many important immune regulators. DAA treatment does not reset the ER stress response or expression of p53 that may contribute to the risk for HCC occurrence after HCV cure by DAA-based antiviral therapy of patients with cirrhosis [218]. DAA-based HCV cure does not reset the intrahepatic ISG expression or stress-related epigenetic signatures, which have been observed to be associated with HCC recurrence [219,220,221]. Cirrhosis is an end-stage liver disease with a multifaceted immune dysfunction due to deterioration of antimicrobial recognition and elimination mechanisms in macrophages along with impaired antigen presentation ability in circulating monocytes [222]. These patients are at significant risk for infection-related mortality. Hepatic adaptive response to stress is at maximum level during liver cirrhosis, which justifies the description of cirrhosis as the world’s most common immunodeficiency syndrome [223]. The activation of stress-related CMA plays a critical role in HCC development and immune dysfunction in cirrhotic patients. Approaches to ameliorate cellular stress could provide a better therapeutic modality for preventing liver disease progression. 

## 7. Treatment of Liver Cirrhosis and Cancer Targeting Autophagy 

The hepatic adaptive response to virus-associated multifaceted stress leads to autophagy regulation and HCC development. Ideally, the virus and ER stress/UPR are the valid frontline targets for therapeutic development to prevent HCC. The successful treatment of HCV has already shown to decrease the incidence of liver cirrhosis and HCC. The HCV-cured patient population who will have the risk of persistent liver disease related to alcoholic and non-alcoholic fatty liver disease is expected to grow exponentially in the coming year. As discussed in the review, the hepatic adaptive response to cellular stress occurs through stress kinases and UPRs. The next logical step will be to develop therapeutic strategies to decrease the adaptive cellular response to the virus infection. Our understanding of the adaptive cellular response to stress and cancer development is not clear. Therefore, it will be wise to understand how adaptive hepatic response to virus-associated stress promotes tumor development using a model system that more closely resembles human disease. The mechanisms of cell death and cell survival pathway regulation during excessive stress that sets the stage of cancer development need to be determined experimentally. Such insights will contribute to the development of more accurate targeted therapy for reducing the cellular stress and cancer incidence at an early stage. In this context, the interaction among the UPR axis, particularly the PERK-eIF2A-ATF4-CHOP-GADD34 axis and Nrf2 activation that regulate cell survival and autophagy, remain the most effective target for the treatment of early-stage HCC [224]. 

The next viable goal is to target the autophagy pathway to inhibit cancer. Small molecule inhibitors targeting the mTOR1 and lysosome have already been in clinical trial development for cancer treatment [225,226]. At present, our understanding of autophagy regulation by cellular stress that leads to HCC is not clear. More publication supports that the impaired autophagy is associated with HCC. Some papers go against this hypothesis. The impaired autophagy leading to cancer development has not been consistent in other cancer models. Likely, adaptive cellular response to biotic and abiotic stress regulates autophagy differently. Therefore, the development of some tumors still needs functional autophagy. HCC growth requires impaired autophagy with high CMA and microautophagy. Understanding how stress-mediated autophagy regulation through CMA activation leads to HCC may also generate the number of therapeutic targets for HCC. Those include Nrf2-inhibitors, LAMP2A, lysosome inhibitors (HCQ), Mdm2 inhibitors (Nutlin3), and inhibitors for PERK, ATF6, IRE1, and GRP78. At present small molecule inhibitors targeting PERK, IRE1, ATF6, NRF2, and eIF2α are currently in clinical development, which can be used to reduce cellular stress to test their role in cancer initiation and growth [227,228,229,230,231,232]. 

## 8. Summary and Conclusions

Although chronic HCV infection is a curable disease, the mechanism of how HCV causes such a high rate of liver cancer is unclear. This review provides the molecular basis of autophagy switching in which tumor suppressor autophagy is converted to tumor-promoting form. Since ER acts as an essential regulator of hepatocyte function, the review provides updated information on how excessive ER stress response generated during chronic HCV infection promotes cell survival pathways that link to HCC development. We begin by explaining multifaceted stress accumulated during HCV replication in the ER, the largest membranous network in the hepatocytes. Infected hepatocytes are provoked by virus-associated stress that leads to the selection of autophagy as a significant cellular process promoting virus-host interaction during chronic infection. The autophagy compensation provided a significant cell survival pathway in chronic HCV infection for hepatocytes threatened by severe virus-related stress, which occurs through an NRF2-dependent manner. The review provided a molecular explanation by which robust stress response accumulated in the cirrhotic liver dampens the natural defense mechanisms of the liver leading to loss of the tumor suppressors, loss of innate immunity, and development of HCC in the cirrhotic liver. The mechanism of autophagy switching during exaggerated UPR response opens up new perspectives for the development of targeted molecular treatment approaches for HCC. This information should allow active biomarker development and a novel approach to treat liver cancer. We propose that the cancer mechanisms related to HCV, a viral agent that resides in the cytoplasm, will facilitate understanding of the liver cancer associated with non-viral etiologies as well.
